# Analysis of LINE1 Retrotransposons in Huntington’s Disease

**DOI:** 10.3389/fncel.2021.743797

**Published:** 2022-01-14

**Authors:** Lavinia Floreani, Federico Ansaloni, Damiano Mangoni, Elena Agostoni, Remo Sanges, Francesca Persichetti, Stefano Gustincich

**Affiliations:** ^1^Area of Neuroscience, Scuola Internazionale Superiore di Studi Avanzati (SISSA), Trieste, Italy; ^2^Central RNA Laboratory, Istituto Italiano di Tecnologia—IIT, Genova, Italy; ^3^Department of Health Sciences, University of Piemonte Orientale “ A. Avogadro,” Novara, Italy

**Keywords:** Huntington’s disease, transposable element (TE), LINE1, HD mouse models, retrotransposition

## Abstract

Transposable elements (TEs) are mobile genetic elements that made up about half the human genome. Among them, the autonomous non-LTR retrotransposon long interspersed nuclear element-1 (L1) is the only currently active TE in mammals and covers about 17% of the mammalian genome. L1s exert their function as structural elements in the genome, as transcribed RNAs to influence chromatin structure and as retrotransposed elements to shape genomic variation in somatic cells. L1s activity has been shown altered in several diseases of the nervous system. Huntington disease (HD) is a dominantly inherited neurodegenerative disorder caused by an expansion of a CAG repeat in the *HTT* gene which leads to a gradual loss of neurons most prominently in the striatum and, to a lesser extent, in cortical brain regions. The length of the expanded CAG tract is related to age at disease onset, with longer repeats leading to earlier onset. Here we carried out bioinformatic analysis of public RNA-seq data of a panel of HD mouse models showing that a decrease of L1 RNA expression recapitulates two hallmarks of the disease: it correlates to CAG repeat length and it occurs in the striatum, the site of neurodegeneration. Results were then experimentally validated in *Htt*^*Q*111^ knock-in mice. The expression of L1-encoded proteins was independent from L1 RNA levels and differentially regulated in time and tissues. The pattern of expression L1 RNAs in human HD post-mortem brains showed similarity to mouse models of the disease. This work suggests the need for further study of L1s in HD and adds support to the current hypothesis that dysregulation of TEs may be involved in neurodegenerative diseases.

## Introduction

Huntington’s disease (HD) is a dominantly inherited neurodegenerative disorder, characterized by progressive motor impairment, cognitive decline and psychiatric disturbances. The most prevalent manifestation of the disease is the selective loss of medium-sized spiny neurons of the striatum. In the later stage of the disease, pathologic alterations have been described in other brain regions, including the cerebral cortex (Vonsattel et al., 1985). The HD mutation alters a polymorphic CAG trinucleotide repeat in the first exon of *HTT*, the gene encoding for huntingtin (Huntington’s Disease Collaborative Research Group, 1993). The number of CAG repeats varies between 6 and 35 units on normal chromosomes, whereas on *HD* chromosomes the repeat is expanded above the pathological threshold of 36 CAGs ranging as high as 150 or more. Age at onset is inversely correlated to CAG length and modified by somatic expansion of the trinucleotide repeat in critical target cells (Genetic Modifiers of Huntington’s Disease (GeM-Hd) Consortium, 2019). The mutant protein presents an expanded polyglutamine (polyQ) tract at the N-terminal that confers the protein the tendency to aggregate. Neuronal intranuclear inclusions (NIIs) and neuropil aggregates that stain positive for huntingtin are histopathological markers of the disease (DiFiglia et al., 1997; Gutekunst et al., 1999). In HD target neurons, the trigger event driven by mutant huntingtin (mHTT) is likely to occur many years before the first signs of neurodegeneration. mHTT expression is associated with DNA lesions and the activation of the DNA damage response (DDR) pathway (Bae et al., 2005; Anne et al., 2007; Illuzzi et al., 2009). It triggers epigenetic-chromatin deregulation at least in part through its ability to facilitate polycomb repressive complex 2 (PRC2) activity (Seong et al., 2010; Ng et al., 2013; Biagioli et al., 2015; Bassi et al., 2017). The disease cascade that leads to death of neuronal cells, involves several pathways affecting neurogenesis (Molero et al., 2009; Nguyen et al., 2013), transcriptional regulation (Seredenina and Luthi-Carter, 2012), protein degradation, glutamate-mediated excitotoxicity, mitochondrial dysfunction and inflammation (Ross and Tabrizi, 2011).

HD mouse models have provided precious tools to unveil molecular mechanisms of mHTT-dependent neurodegeneration, identify potential pharmacological targets and test new treatments at pre-clinical stage. Several mouse lines have been generated to date with different approaches in genetic design and distinct ability to recapitulate the crucial features of the human disease such as dominant inheritance, length-repeat dependent age at onset and striatal specificity of neuronal dysfunction (Mangiarini et al., 1996; White et al., 1997; Pouladi et al., 2013). Among them, full-length mHTT rodent models have been established by knocking-in expanded CAG repeat tracts into the endogenous mouse *Htt* gene locus. In this context, a recent RNA-seq study profiled the striatum and cortex of heterozygous *Htt* knock-in mice with six different CAG lengths and conceptually encoding different polyQ stretches. By analyzing mice at different ages, 13 striatal and 5 cortical gene expression modules were found highly correlated to CAG length and age (Langfelder et al., 2016).

Extensive characterization of *Htt*^*Q*111^ mice has been carried out in several laboratories. They display molecular and behavioral phenotypes recapitulating many features of the human disease in a time-dependent fashion (Fossale et al., 2002; Wheeler et al., 2002; Gines et al., 2003; Lloret et al., 2006; Lynch et al., 2007; Carnemolla et al., 2009; Giralt et al., 2012; Hölter et al., 2013; Agostoni et al., 2016). In the first months of life, molecular changes include the activation of DDR and the induction of ribosome biogenesis regulator 1 (*Rrs1*) expression, a nucleolar protein involved in rRNA biogenesis and endoplasmic reticulum stress. At 3 months of age (3 mo), mHTT accumulates in the nucleus of medium spiny neurons. At 12 months of age (12 mo), striatal cells display NIIs together with changes in the expression of a large number of genes. At 24 months of age (24 mo), neurodegeneration of medium spiny neurons and reactive gliosis are evident.

Despite the large amount of information, the detailed understanding of the molecular basis of HD pathogenesis remains incomplete and a treatment for this devastating disease is still not available. It is therefore important to keep investigating potential previously unnoticed pathways that may be altered in HD and target of therapeutic treatments.

In the last few years interesting correlations have been observed between neurological diseases and transposon activation. Transposable elements (TEs) are mobile genetic elements that constitute a significant fraction of eukaryotic genomes (Huang et al., 2012). Long Interspersed Nuclear Elements 1 (L1s) are the most abundant TEs (∼17% of the human genome) and the only transposon class retaining the ability to mobilize autonomously in human (Goodier et al., 2001; Ostertag and Kazazian, 2001). They take advantage of a “copy and paste” mechanism where a full-length sequence gives rise to a L1 RNA intermediate that is reverse-transcribed and “pasted” into a new genomic locus. Retrotransposition is mediated by L1-encoded ORF1 and ORF2 proteins (ORF1p and ORF2p) generating mostly 5′-truncated L1s that are unable to re-mobilize. ORF1p encodes for a nucleic acid chaperone while ORF2p for endonuclease and reverse transcriptase activities. To prevent potential deleterious effects of L1 abnormal activity, cells have developed several mechanisms to safeguard and fine-tune L1 retrotransposition. These include DNA methylation, transcriptional repression and L1s RNA degradation through the activity of the PIWI/piRNA pathway (Ozata et al., 2019). Recently, attention has been focused on the functional role of L1s independent from retrotransposition (Faulkner et al., 2009). The majority of L1 RNAs is retained in the nucleus regulating chromatin structure and participating in transcriptional control (Blaudin de Thé et al., 2018). L1 RNA expression and mobilization must therefore be considered two independent events under distinct regulatory pathways and with different functional outcomes (Jachowicz et al., 2017; Wang et al., 2018).

More than 100,000 L1 copies have been identified in the mouse genome (Goodier et al., 2001). Full-length sequences may vary between 6 up to 8 kb (Sookdeo et al., 2013). The presence of a variable region located in the 5′UTR, containing monomers of ∼200 bp, allowed the identification of different families whose active members are *A*, *Gf*, and *Tf*. The *A* family includes about 6,500 full-length copies, of which ∼900 with intact ORF1 and ORF2 sequences (Severynse et al., 1992; Adey et al., 1994; DeBerardinis and Kazazian, 1999; Jachowicz and Torres-Padilla, 2016). The most recent *Tf* and *Gf* L1 families account for the majority of transcribed L1s, including 1800 *Tf* out of 3000 full-length members (Naas et al., 1998) and 400 *Gf* among 1500 (Goodier et al., 2001). Mounting evidence shows that L1s are active during neurogenesis in mammals and somatic mobilization of TEs has been observed in mouse and human brain regions giving rise to mosaicism (Muotri et al., 2005; Coufal et al., 2009; Baillie et al., 2011; Evrony et al., 2012). L1s seem to preferentially insert in the chromatin of neuronally expressed genes although the extent and the functional role of TEs mobilization in brain physiology remain unclear (Upton et al., 2015; Evrony et al., 2016; Paquola et al., 2017).

Emerging evidence suggests an association between unregulated activation of TEs and diseases of the nervous system (McConnell et al., 2017; Jacob-Hirsch et al., 2018). Pathological TE activation has been observed in animal or cellular models and/or in human tissues of several diseases including Rett syndrome (Muotri et al., 2010), ataxia telangiectasia (Coufal et al., 2011), macular degeneration (Kaneko et al., 2011), prion diseases (Lathe and Harris, 2009), amyotrophic lateral sclerosis (ALS) (Douville et al., 2011; Li et al., 2012; Krug et al., 2017; Tam et al., 2019), Alzheimer’s disease (Guo et al., 2018; Sun et al., 2018), Parkinson’s disease (Blaudin de Thé et al., 2018) and schizophrenia (Bundo et al., 2014).

Recently, a preliminary characterization of R6/2 mice, a transgenic model expressing exon 1 of human N-mut HTT containing 150 poly-Q repeats, showed an increase of L1s expression and mobilization (Tan et al., 2018).

Here we carry out RNA-seq analysis from public data of a panel of knock-in HD mouse models to investigate whether L1 expression changes in diseased brains. We quantify L1 RNA levels in correlation with CAG repeat length and with brain areas to monitor striatal specific changes, that both represent hallmarks of the human disease. Results are then experimentally validated in *Htt*^*Q*111^ knock-in mice together with the analysis of L1-ORFs proteins expression. Finally, RNA-seq analysis from public data of human HD post-mortem brains unveils commonalities with L1s pattern of expression in mouse models.

## Materials and Methods

### Bioinformatic Analysis of RNA-Seq Data in Mice

We took advantage of RNAseq dataset in Langfelder et al. (2016). This study was carried out in the striatum and cortex at 2, 6, and 10 months old (mo) heterozygous *Htt* knock-in mice of three different CAG lengths (denoted Q: Q20, Q111 and Q175). The dataset was downloaded from ENA-EBI database^1^ (Supplementary Table 1). Fasta file containing the nucleotide (nt) sequences of all the mouse TE consensus sequences were downloaded from RepBase database (Bao et al., 2015). A total of 144 paired end (PE) samples (2 tissues, 3 genotypes, 3 ages and 8 replicates) were analyzed. We aligned the PE reads to all the TE consensus sequences retrieved from the RepBase database using bwa mem version 0.7.15 (Li and Durbin, 2009) with default parameters. Using samtools version 1.3.1 (Li et al., 2009), we selected only read pairs properly mapped (–f 0 × 0002 parameter). Using bedtools coverage version 2.26.0 (Quinlan and Hall, 2010), we counted the reads mapping on each analyzed TE. No potentially duplicate reads were discarded. The number of reads mapping on every element was normalized to the total number of reads of the sample. Prior to the identification of differentially expressed TEs, TEs showing low level of expression were removed from the analysis (selected only the TEs displaying at least 15 mapped reads in at least 8 samples). Then, for each remaining TE consensus, an independent 2-group *t*-test was carried out on the expression levels for the 3 different ages (2, 6, and 10 mo) using Q20 mice as control and Q111 and Q175 as diseased samples, in striatum and cortex separately (Supplementary Tables 2–4). The *t*-test *p*-values were adjusted using false discovery rate correction (FDR—Benjamini and Hochberg). The final plots were generated using R ggplot2 library (Wickham, 2016).

### Mice

Cortex and striatum tissue samples were dissected from 3, 12, and 24 months old (mo) heterozygous *Htt* knock-in mice (*Htt*^*Q*111^ denoted as HD: Q7/Q111) and wild-type (denoted as WT: Q7/Q7) littermate in C57BL/6 background (Lloret et al., 2006). Animals were kindly provided by M. MacDonald (Massachusetts General Hospital, Boston, MA, United States). Animal care, handling and subsequent procedures were performed in accordance with the European Community Council Directive of November 24, 1986 (86/609EEC) and following SISSA Ethical Committee permissions.

### Total RNA Extraction

Total RNA was extracted from 50 mg of bulk tissue using Trizol reagent according to manufacturer’s instructions (Ambion). To remove any residual DNA, RNA samples were treated with DNAse I (Ambion) using 2 U every 10 μg of RNA added with SUPERaseRNAse inhibitor (Invitrogen) in a reaction volume of 50 μL and incubated at 37°C for 30 min. After treatment, enzyme was inactivated and RNA was purified using Cleanup RNeasy^®^ Mini kit according to manufacturer’s instructions (QIAGEN). The RNA concentration and purity were determined by spectrophotometric measurement using NanoDrop 2000 (Thermo Fisher Scientific) and by running denaturing formaldehyde agarose gel.

### qRT-PCR

In reverse transcription (RT) reactions, 0.5 μg of total RNA were used as template. Reverse transcription was performed using iScript cDNA Synthesis kit according to manufacturer’s instructions (Biorad). For each sample, both RT^+^ and RT^–^ reactions were run by adding or lacking reverse transcriptase enzyme. Quantitative real-time PCR (qPCR) was performed using CFX96 Touch*™* Real-Time PCR Detection System (Biorad) machine using iQ*™* Multiplex Powermix (Biorad). qPCR experiments for L1 elements were performed in duplex using FAM-labeled *Taqman* probes against the specific 5′UTR L1 subfamily together with VIC-labeled *Taqman* probe against housekeeping gene UbC. PCR amplification was performed in a final volume of 20 μL using the following parameters: (1) 95°C for 20 s, (2) 95°C for 10 s, (3) 59°C for 30 s. Steps (2) and (3) were repeated 40 times. In qPCR experiments, we loaded both RT^+^ and RT^–^ samples to control residual genomic DNA contamination. We accepted a minimum of 6 cycles of difference between RT^–^ and RT^+^ samples for the 5′UTR target sequence. For relative quantification of the transcripts, the 2^(–ΔΔ*Ct*)^ method was used (Livak and Schmittgen, 2001). For each assay, at least three independent qPCR technical replicas were performed on all samples. Primers and probes used are listed in Supplementary Table 5.

### Statistical Analysis and Graphs

In Scatter plots, each dot represents the mean value of at three independent qPCR replica. Mean value is shown with red horizontal bar. Error bars represent standard error. Statistical analyses were performed by means of two tailed, Mann Whitney non-parametric test, taking advantage of GraphPad Prism 5 Statistics Toolbox. * *p*-value < 0.05, ^**^ *p*-value < 0.01.

### Western Blot

For ORF1p and ORF2p analyses, total protein lysates from cerebral cortex and striatum were obtained by incubation for 30 min on ice, using RIPA buffer (10 mMTris-HCl, 140 mMNaCl, 1 mM EDTA, 1% Triton X-100, 0.1% sodium deoxycholate, 0.1% SDS) supplemented with 1% v/v Protease Inhibitor Cocktail (cat. P8340, Sigma-Aldrich). Genomic DNA was sheared by sonication (three cycles of 5′′ each on ice). Lysates were clarified by centrifugation (10,000 × g, 30′ at 4°C) and supernatants quantified by Pierce BCA protein assay kit (cat. 23225, Thermo Fisher Scientific). Ten microgram/samples were denatured in 4X Laemmli’s sample buffer (240 Tris-HCl pH 6.8, 40% glycerol, 8% SDS, 0.04% bromophenol blue, 5% beta-mercaptoethanol), boiled for 5′ at 95°C and loaded on a 10% acrylamide Bis-Tris gel. Proteins were transferred to nitrocellulose membrane. Membranes were blocked with 5% non-fat milk in Tris Buffer Saline Tween20 (TBST) for 1 h at room temperature and then incubated at 4°C overnight with primary antibody. Proteins were then detected using HRP-conjugated antibodies. Membrane was developed using SuperSignal West Pico PLUS Chemoluminescence Substrate (Thermo Fisher Scientific). Primary antibodies were: mouse monoclonal IgG anti-ORF1p (5 μg/mL) and mouse monoclonal IgM anti-ORF2p (10 μg/mL), anti b-actin (0.5 μg/mL, Sigma Aldrich). Anti-ORF1p and anti-ORF2p antibodies were kindly provided by Gerald Schumann, Paul Ehrlich Institute, Langen (Germany) (Lucchinetti et al., 2006; Kirilyuk et al., 2008).

### Bioinformatic Analysis of RNA-Seq Data in Human Post-mortem Brains

RNA sequencing (RNAseq) data from Labadorf et al. (2015) was analyzed to study the expression of the retrotransposon LINE-1 in BA9 region of post mortem human brains. Globally, we analyzed 69 paired-end (PE) samples composed by 49 healthy controls and 20 HD. For the analysis we used a stringent bioinformatics pipeline as described in Ansaloni et al. (2019). Briefly, we downloaded a fasta file containing the nucleotides (nt) sequences of all the human repeats (except simple repeats) from RepBase database (Bao et al., 2015) and a fasta file containing both coding and non-coding transcripts (GRCh38) from Ensembl (Zerbino et al., 2018). We then map read pairs on a reference transcriptome built merging RepBase transposon and Ensembl transcriptome using STAR version 2.6.0c (Dobin et al., 2013) and assigning primary alignment flag to all the alignments with the best score (–outSAMprimaryFlag AllBestScore). Using samtools version 1.3.1 (Li et al., 2009), we selected only mappings flagged as primary (–F 0 × 100 parameter), without discarding potentially duplicate reads. To avoid selection of reads mapping on TE fragments embedded in coding and/or long non-coding transcripts we discarded best-scoring alignments containing read pairs mapping both on transposon and transcriptome using scripts in python3 and Picard FilterSamReads tool.^2^ Using bedtools coverage version 2.26.0 (Quinlan and Hall, 2010) we counted the reads passing all the filters of each of the 69 samples relatively to L1HS elements. Each count was normalized on the total number of reads of the sample and an independent 2-group *t*-test was performed on the normalized read counts. The final plots were generated using R ggplot2 library (Wickham, 2016).

## Results

### L1 RNA Expression Decreases in Huntington Disease Mice With Increased Polyq Lengths

To investigate whether L1 expression is altered in HD mouse models, we took advantage of the RNA sequencing (RNAseq) dataset by Langfelder et al. (2016). In their study, a gene expression analysis was carried out on the striatum and cortex of 2-, 6- and 10-mo old heterozygous *Htt* knock-in mice of three different CAG lengths indicated as Q20, Q111, and Q175. This dataset is important to explore whether L1s expression is altered in diseased mice and whether these changes recapitulate the hallmarks of human disease: its dependency on poly(Qs) length, its brain area specificity and its correlation with age.

**FIGURE 1:**
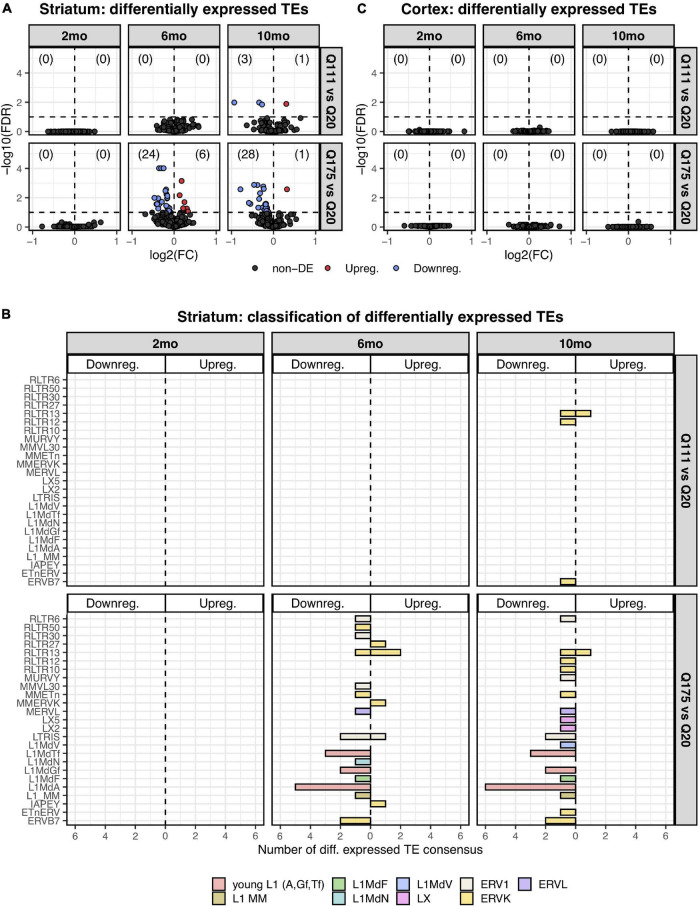
Differentially expressed TEs. **(A)** Volcano plot showing the murine TE consensus sequences differentially expressed in the striatum of 2-, 6-, and 10-mo mice in Q111 and Q175 genotypes compared to control (Q20). Blue color indicates the TE consensus sequences that result downregulated in each tested comparison (Q111 vs. Q20 and Q175 vs. Q20), red indicates the upregulated TE consensus sequences whereas in black are depicted the TE consensus sequences that do not result differentially expressed (non-DE). Each group is composed by 8 biological replicates (*n* = 8). **(B)** Bar plot reporting the number of differentially expressed TE consensus sequences for each differentially expressed TE subfamily. L1 *A*, *Gf* and *Tf* are classified as “young L1.” **(C)** Differentially expressed TEs in the cortex of 2-, 6-, and 10-mo mice in Q111 and Q175 genotypes compared to control (Q20). Each group is composed by 8 biological replicates (*n* = 8).

**FIGURE 2:**
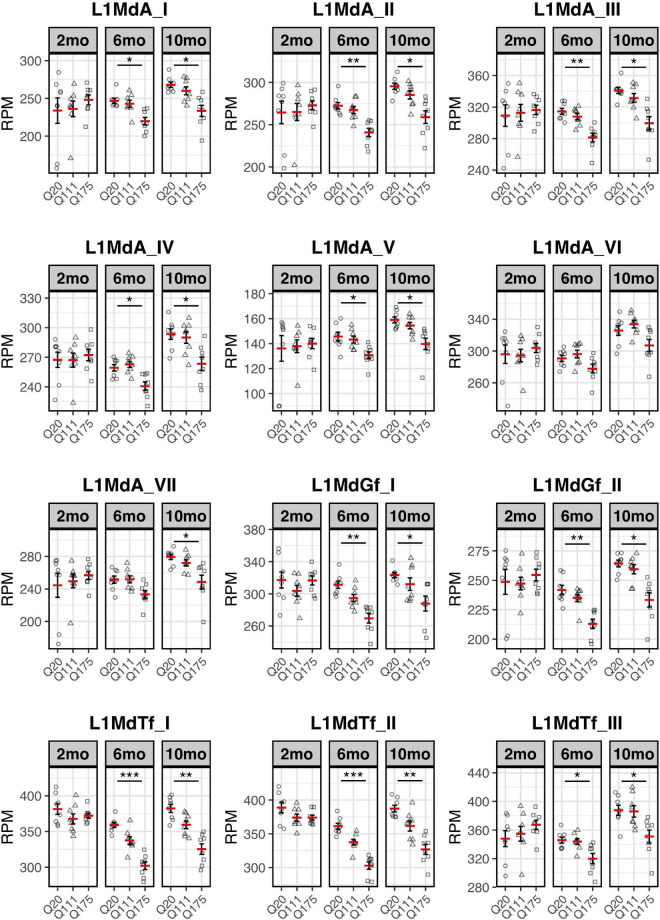
Expression of the 7 L1-A (L1MdA), 2 L1-Gf (L1MdGf), and 3 L1-Tf (L1MdTf) in the mouse striatum. L1 expression is reported as number of mapped reads normalized on the total number of reads of each sample (RPM) for Q20 (control) (circle), Q111 (triangle) and Q175 (square) in 2-month-old mice (2 mo—left panel), 6-month-old mice (6 mo—middle panel), and 10-month-old mice (10 mo—right panel). Horizontal red segment represents mean, error bars report mean ± sem (standard error of the mean). *FDR < 0.05, **FDR < 0.01, ***FDR < 0.001. Each group is composed by 8 biological replicates (*n* = 8).

Paired end read from 144 samples (Supplementary Table 1) were aligned on all the murine RepBase TE consensus sequences (see section “Materials and Methods”). We counted reads mapping on each TE consensus normalizing the resulting values to the number of reads of each sample. An independent 2-group *t*-test was carried out on the expression levels for both striatum and cortex for each TE class, for the 3 different ages (2-, 6-, and 10-mo) using Q20 as control and Q111 and Q175 as diseased samples (Supplementary Tables 2–4). As shown in Figure 1A, a significant decrease (FDR < 0.1) in the expression levels of 24 and 28 TEs was evident in the striatum of, respectively, 6− and 10-mo old Q175 mice compared to age matching Q20 controls. The majority of these significantly down-regulated TEs were L1 elements (13 out of 24 and 16 out of 28) (Figure 1B). Most of them belong to evolutionary young L1 subfamilies such as *A*, *Tf* and *Gf* (10 out of 13 and 11 out of 16). Importantly, a general trend was observed linking the decrease of expression to the number of Qs for all the *A*, *Tf* and *Gf* L1 elements (Figure 2). On the contrary, no statistically significant changes of L1s expression were observed in the cortex of diseased mice at all ages (Figure 1C and Supplementary Figure 1).

These results support the hypothesis that L1 expression is altered in HD and that these changes recapitulate crucial hallmarks of the disease, including their dependency on poly(Qs) length and age as well as their specificity for the injured brain region.

### Experimental Validation of Changes in L1 RNA Expression in Wild-Type and Huntington Disease Knock-in Mice

**FIGURE 3:**
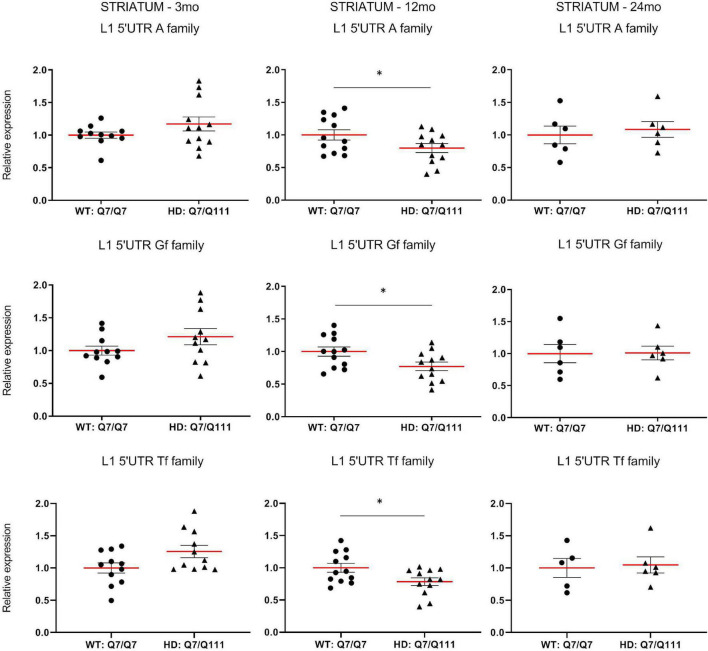
Expression of full-length L1s mRNA in postnatal striatum of HD and WT mice. Relative quantification of L1 5′UTR levels in the striatum of WT: Q7/Q7 and HD: Q7/Q111 mice at 3, 12, and 24 mo of age. Relative L1 5′UTR expression levels are normalized with the housekeeping gene UbC. Each dot results from 3 independent qPCR replica on each sample. Mean value is shown with red bar. Error bars indicate SEM. *P < 0.05 resulting from *Mann Whitney* unpaired test. Groups for 3 and 12 mo mice are composed by 12 biological replicates (*n* = 12). Groups for 24 mo mice are composed by 6 biological replicates (*n* = 6).

**FIGURE 4:**
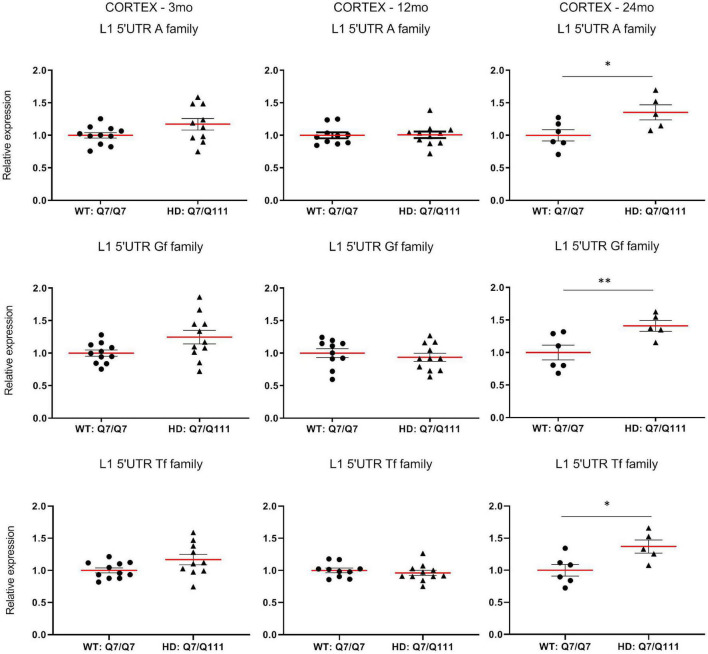
Expression of full-length L1s mRNA in postnatal cortex of HD and WT mice. Relative quantification of L1 5′UTR mRNA levels in the cortex of WT: Q7/Q7 and HD: Q7/Q111 mice at 3, 12, and 24 mo of age. Relative L1 5′UTR expression levels are normalized with the housekeeping gene UbC. Each dot results from 3 independent qPCR replica on each sample. Mean value is shown with red bar. Error bars indicate SEM. *P < 0.05, ^**^P < 0.01 resulting from *Mann Whitney* unpaired test. Groups for 3 and 12 mo mice are composed by 12 biological replicates (*n* = 12). Groups for 24 mo mice are composed by 6 biological replicates (*n* = 6).

To experimentally validate HD-dependent changes in the expression of L1 elements, we first designed specific *Taqman* qPCR assays for full-length *A*, *Tf* and *Gf* families (Supplementary Figure 2). Then we compared L1 expression of heterozigous *Htt*^*Q*111^ knock-in mice (denoted: HD: Q7/Q111) to wild-type littermate controls (denoted: WT: Q7/Q7) at 3−, 12−, and 24-mo of age. All three L1 families showed a statistically significant decrease of expression in the striatum of HD: Q7/Q111 mice at 12 months of age (Figure 3). No differences were observed in all the remaining time points. On the contrary, a statistically significant increase of full-length L1 expression for all 3 families was observed in the cortex of 24 mo HD: Q7/Q111 mice (Figure 4).

### ORF1p and ORF2p Are Differentially Expressed in Huntington Disease Mice

Full-length L1 mRNA is a bicistronic transcript that encode for ORF1 and ORF2 proteins (ORF1p and ORF2p). ORF1p is an RNA binding protein with chaperone activity, whereas ORF2p has both endonuclease and reverse transcriptase activities. Together, these two proteins provide the molecular machinery for the mobilization of L1 and other cellular RNAs.

**FIGURE 5:**
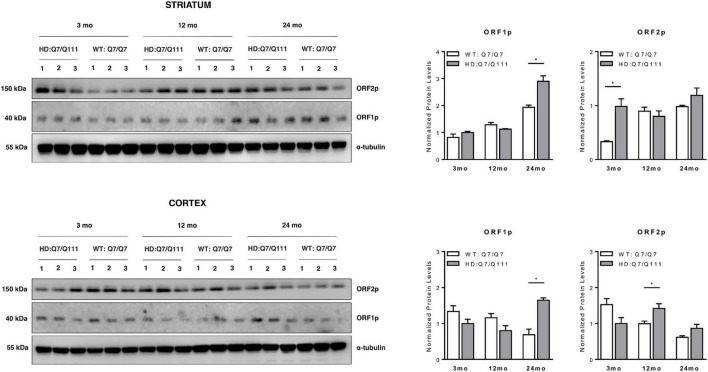
ORF1p and ORF2p expression in postnatal striatum and cortex of HD and WT mice. Representative western blot showing ORF1p and ORF2p levels in protein lysates of striatum (A) and cortex (B) of 3 WT: Q7/Q7 and 3 HD: Q7/Q111 mice at 3, 12 and 24 mo of age. α-tubulin is used as loading control. Bar plots represent normalized protein levels resulting from 3 replica. At each developmental stage, protein levels are compared between WT vs. HD mice. Error bars indicate SEM. *P < 0.05, resulting from paired *t*-test.

We next investigated whether full-length L1 mRNAs expressed in the brain of adult mice may lead to endogenous expression of L1-encoded proteins and whether they are differentially expressed in HD vs. WT mice. Western Blot analysis revealed that both ORF1p and ORF2p are endogenously expressed in the striatum and cerebral cortex of adult HD: Q7/Q111 and WT: Q7/Q7 mice of 3−, 12−, and 24-mo of age (Figure 5). Data shows that striatum and cerebral cortex displayed distinctive expression patterns of L1-encoded proteins. Furthermore, within the same tissue, protein levels of ORF1p and ORF2p did not correlate proportionally one to one another. In WT mice we observed an age-dependent increase of both ORF1p and ORF2p expression in the striatum while in the cortex the trend was the opposite, although not statistically significant. Interestingly, a statistically significant increase in ORF1p expression was observed in both striatum and cortex of 24-mo old HD: Q7/Q111 mice. ORF2p expression levels were significantly increased in HD: Q7/Q111 mice at 3-mo in the striatum and at 12-mo in the cortex.

### L1s RNA Expression Decreases in Human Huntington Disease Post-mortem Brains

To investigate whether the differential expression of L1s in HD mouse models occurs also in human post-mortem brains, transcriptomic data from Labadorf et al. (2015) was analyzed. This data set includes 20 HD and 49 neuropathologically normal individuals. HD individuals present a range from 25 to 63 years as age of onset and from 41 to 51 CAG repeats length. Since interpretation of gene expression experiments in post-mortem striatum is hampered by neuronal loss up to 90% in HD brains, mRNA profiling was carried out on prefrontal cortex Brodmann area 9. It is well-established that this region is involved in HD pathogenesis but suffers substantially less neuronal death than striatum. By taking advantage of a custom bioinformatic pipeline (Ansaloni et al., 2019), 69 PE samples were analyzed. To this purpose, a reference dataset of human TE sequences was obtained as described in “Materials and Methods.” By using bedtools coverage (Quinlan and Hall, 2010), selected reads of each of the 69 samples were counted as mapped on the L1HS element. The total score was then normalized on the number of reads of the sample. An independent 2-group *t*-test was then carried out on the normalized read counts.

**FIGURE 6:**
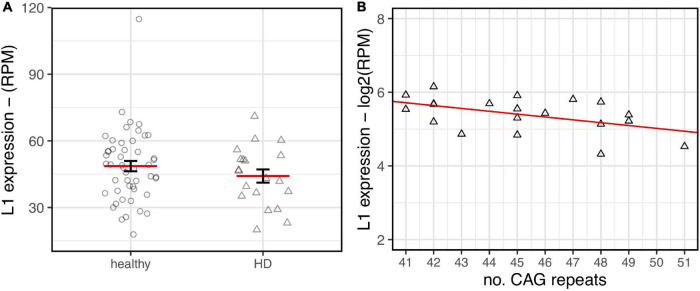
L1HS expression in HD post-mortem human brains. RNA-seq data from healthy samples (circle, *n* = 49) and HD patients (triangle, *n* = 20) have been analyzed. **(A)** A decreasing trend of expression in HD brains is highlighted although lacking statistical significance (*p*-value = 0.24). **(B)** Normalized L1 expression values (*y*-axis) are associated to the number of CAG repeats (*x*-axis) for human HD samples. The regression line shows a decreasing trend that reaches statistical significance (*R*^2^ = 0.24–*p*-value = 0.029).

As shown in Figure 6, a lower expression of L1s RNA in HD human brains was observed although not statistically significant. Interestingly, despite the low range of CAG repeats numbers in the HD samples, a statistically significant inverse correlation between CAG length and L1 expression was evident. These preliminary results from human post-mortem brain support our observations made in HD mouse models.

## Discussion

### L1s Expression in Huntington Disease Mice Models and Post-mortem Brains

The decrease of L1 RNA expression in HD mouse models follows two hallmarks of the disease: (i) It is dependent on CAG repeat length; (ii). It is occurring in the striatum, the site of neurodegeneration. Measuring RNA-seq reads mapping to the more active L1 families in mice, we found that there is a statistically significant decrease of expression of L1s in Q175 mice and a similar trend in Q111. The decrease was not occurring at 3 mo old Q175 mice while it was exacerbated with aging, from 6 to 10 mo. Furthermore, it took place exclusively in the striatum. These results were then experimentally validated for the most part by qRT-PCR in HD: Q7/Q111 mice. Some differences in expression may be accounted for by the details of the two approaches. Expressed L1 RNAs present a heterogeneous repertory of transcripts including full-length and 5′ truncated RNAs. While special attention has been devoted to discard embedded L1s in protein coding mRNAs and non-coding RNAs, the bioinformatic pipeline carried out on the public RNA-seq dataset counts reads along the entire L1 transcripts, including the expression of truncated retrotransposons. On the other hand, qRT-PCR experiments detected the expression of mainly full-length transcripts. Two months of difference in the age of analysis could attenuate the extent of gene expression changes of L1s RNA in younger animals.

Importantly, a decrease trend of L1 expression was unveiled in RNA-seq data of HD post-mortem brain where a statistically significant association with CAG repeat length number was shown.

### Candidate Mechanisms of L1s Expression Changes in Huntington Disease

The first evidence of L1 differential expression in the striatum of HD: Q7/Q111 mice occurs at 12 mo of age, before the appearance of behavioral changes. At this stage, NIIs are accumulating in medium spiny striatal cells and a concomitant dysregulation of hundreds of genes takes place in the presence of extensive DNA damage. Some potential mechanisms may account for L1 expression attenuation. Altered methylation of DNA and histone tails has been reported in the striatum of HD mice at 12 mo of age (Bassi et al., 2017). Interestingly, aberrant interaction of mHTT with MeCP2 or PRC2 modulates their methylation activity on target genes in a polyglutamine-dependent fashion (Seong et al., 2010; McFarland et al., 2014). In particular, the formation of the mHTT-MeCP2 complex contributes to transcriptional inhibition in HD. Both MeCP2 and PRC2 are well known regulator of L1 transcription (Muotri et al., 2010; Padeken et al., 2015; Upton et al., 2015) suggesting their potential role in L1 expression control in HD mice and post-mortem brains. In addition, induction of inhibitory transcriptional repressors or inhibition of transcriptional activators of L1s could be involved. Given the long list of transcription factors shown to regulate L1 expression or dysregulated in HD (Labadorf et al., 2015, 2017), several candidates deserve further attention and experimental validation.

The piRNA pathway is one of the main mechanisms of transcriptional and post-transcriptional L1 regulation. First discovered in *Drosophila melanogaster*, the murine homologs of fly PIWI proteins, MIWI and MILI, as well as piRNAs were recently identified in adult mouse brains (Ghosheh et al., 2016; Nandi et al., 2016), playing crucial roles in promoting *de novo* DNA methylation on L1 promoters (Aravin et al., 2008; Kuramochi-Miyagawa et al., 2008). During this study, the expression of MILI in adult striatum was experimentally validated although no changes in expression in HD mice was observed (*data not shown*). Further analysis of MILI and piRNAs will assess whether this pathway is involved in L1 expression control in HD.

### Analysis of Retrotransposition Machinery in Huntington Disease: Q7/Q111 Mice

The expression of ORF1p and ORF2p in selected tissues and ages has evidenced that there was no correspondence between total L1 RNA amounts and protein levels and that their expressions were independent of each other. Several examples of lack of correlation among total L1 RNA amount, ORF1p and ORF2p levels and mobilization have been reported so far (Jachowicz et al., 2017; Wang et al., 2018). This is probably due to multiple regulatory layers that define the quantity of L1-encoded ribonucleoparticles available for retrotransposition in specific cell types at a given time. Increasing evidence shows that a substantial amount of L1s RNA is nuclear-retained (Liu et al., 2020) where they may function as regulatory long non-coding RNAs. The recent identification of large number of DNA/RNA hybrids at L1s loci may suggest TEs may exert their function *in cis* (Percharde et al., 2018) although evidence show they can also act *in trans* organizing chromatin domains. By their pattern of protein interactions, they may recruit protein complexes to specific regions of the genomes (Lu et al., 2020). ORF1p and ORF2p are encoded by a bicistronic mRNA where ORF2p translation is sustained by an Internal Ribosomal Entry Site (IRES). It is well known that IRES sequences regulate translation in *cis* when CAP-dependent translation is attenuated, as in stress condition, providing a mechanism for ORF1p and ORF2p differential expression. Furthermore, they could be translated by independent mRNA species including 5′ or 3′ truncated L1 RNAs.

ORF1p and ORF2p were indeed found differentially expressed between HD: Q7/Q111 and WT: Q7/Q7 mice at selected ages and brain regions. These results may suggest that the machinery for retrotransposition is in place and that a different pattern of somatic L1s insertions is present in these mice. In a preliminary set of experiments we took advantage of a *Taqman* qPCR assay that amplifies a portion of the ORF2 sequence shared among genomic L1s, recapitulating in rodents experimental approaches previously used on the human genome (Muotri et al., 2005; Coufal et al., 2009). No statistically significant changes in CNVs of L1s were observed (*data not shown*). However, lack of evident changes with this assay on “bulk” DNA does not necessarily mean that no differential retrotransposition has occurred. While estimates of cellular mosaicism in mammals have led to a wide range of potential novel insertions per cell (from 0.2 to 16.3) (Upton et al., 2015; Evrony et al., 2016), they are well below the sensitivity of this assay. In the future, whole genome sequencing of bulk DNA samples at high depth or at single cells level will be necessary to assess truly whether HD mice present an altered rate of retrotransposition.

Interestingly, a general trend of increased expression of L1-encoded proteins correlated with aging in the striatum, culminating with the highest expression of ORF1p at 24 mo in HD: Q7/Q111 mice. In the cortex at 24 mo, both L1 RNAs and ORF1p present a higher expression in HD: Q7/Q111 mice. Considering that at this age, HD: Q7/Q111 mice show extensive neurodegeneration and widespread reactive gliosis (Wheeler et al., 2002), we hypothesize that these results may be due to changes in cellular composition and a consequence of the fact that expressions are measured from the “bulk” tissue. These results can also in part account for apparently contrasting data of L1 expression and mobilization in R6/2 mice, a transgenic HD model expressing exon 1 of human N-mut HTT containing 150 poly-Q repeats (Tan et al., 2018). These mice exhibit an early widespread and generalized degenerative phenotype with rapid onset of symptoms, including motor, cognitive and behavioral abnormalities, weight loss and a reduction in lifespan (Mangiarini et al., 1996). By looking at L1 expression and CNV in 10–12 weeks old mice, an increase in expression and mobilization was found. This pattern is concomitant to previously described neuronal cell loss (Stack et al., 2005), anatomical and functional alterations in microglia (Savage et al., 2020) and increased expression of genes related to gliosis and the immune response (Luthi-Carter et al., 2002; Hodges et al., 2008). These results are therefore compatible with the accepted view that R6/2 mice represent an accelerated model of HD (Brooks et al., 2012; Pouladi et al., 2013). We are currently assessing whether the increased L1 RNA expression observed at 24 mo in the cortex of HD: Q7/Q111 mice is also manifesting in the striatum of older mice and leading to increased mobilization.

## Conclusion

In summary, we show that there is an inverse correlation between L1 expression and CAG repeat length in genetically precise HD mouse models and human post-mortem brains. The decrease of L1 RNA levels occurs specifically in the striatum, the site of neurodegeneration. Given the uncoupling between transcription, translation and mobilization of L1s in HD, several functional outputs could be hypothesized for these changes in expression. Among them, given the recent observation that nuclear-retained L1s RNA plays a fundamental role in transcriptional control and chromatin structure, further study should address the intriguing hypothesis that the decrease of L1s expression participates in the well-established chromatin remodeling and epigenetic dysregulation in HD.

## Data Availability Statement

The original contributions presented in the study are included in the article/Supplementary Material, further inquiries can be directed to the corresponding author/s.

## Ethics Statement

The animal study was reviewed and approved by the Ministry of Health Italy.

## Author Contributions

LF performed the experiments of L1 expression and CNVs and took care of mice breeding. DM carried out the ORF1 and ORF2 analysis. FA performed the bioinformatic analysis of RNA-seq data. EA developed the mouse *Taqman* CNV PCR assays. RS conceived, supervised the bioinformatic analysis, and wrote the manuscript. FP conceived, supervised the study, and wrote the manuscript. SG wrote the manuscript, conceived, and supervised the study. All authors contributed to the article and approved the submitted version.

## Conflict of Interest

The authors declare that the research was conducted in the absence of any commercial or financial relationships that could be construed as a potential conflict of interest.

## Publisher’s Note

All claims expressed in this article are solely those of the authors and do not necessarily represent those of their affiliated organizations, or those of the publisher, the editors and the reviewers. Any product that may be evaluated in this article, or claim that may be made by its manufacturer, is not guaranteed or endorsed by the publisher.

## Footnotes

1
https://www.ebi.ac.uk/ena

2
https://broadinstitute.github.io/picard/

## Supplementary Material

Supplementary Figure 1 Expression of the 7 L1-A (L1MdA), 2 L1-Gf (L1MdGf), and 3 L1-Tf (L1MdTf) in the mouse cortex. L1 expression is reported as number of mapped reads normalized on the total number of reads of each sample (RPM) for Q20 (control) (circle), Q111 (triangle) and Q175 (square) in 2-month-old mice (2 mo—left panel), 6-month-old mice (6 mo—middle panel), and 10-month-old mice (10 mo—right panel). Horizontal red segment represents mean, error bars report mean ± sem (standard error of the mean). *FDR < 0.05, ^**^FDR < 0.01, ^***^FDR < 0.001.

Supplementary Figure 2 Schematic representation of 5′UTR L1 *Taqman* assay on full-length L1s. Purple, yellow and green boxes indicate, respectively, Gf, A and Tf *Taqman* probes, able to detect only full-length L1 mRNAs. The 5′UTR monomers of full-length mouse L1 elements are represented by triangles and the black bar below indicates the position of primers and *Taqman* probes specifically designed for each monomeric L1 region.

Supplementary Table 1 The 18 different samples analyzed are reported in the 1st column. For each of the 2 tissues (cortex and striatum), 3 different genotypes (Q20, Q111, Q175) at 3 different ages (2, 6, 10 months old) have been analyzed. Each sample has 8 replicates. The replicates accession ID are reported in the 2nd column of the table.

Supplementary Table 2 Differentially expressed TEs in mouse striatum—Q111 vs. Q20–10 month-old. TE name, log2 fold-change, FDR-corrected *p*-value and metadata for the Q111 vs. Q20 comparison in the striatum of 10 month-old mice are reported.

Supplementary Table 3 Differentially expressed TEs in mouse striatum—Q175 vs. Q20–6 month-old. TE name, log2 fold-change, FDR-corrected *p*-value and metadata for the Q175 vs. Q20 comparison in the striatum of 6 month-old mice are reported.

Supplementary Table 4 Differentially expressed TEs in mouse striatum—Q175 vs. Q20–10 month-old. TE name, log2 fold-change, FDR-corrected *p*-value and metadata for the Q175 vs. Q20 comparison in the striatum of 10 month-old mice are reported.

Supplementary Table 5 List of primer and probes of L1 5′UTR A, Gf, Tf expression assays.
