# Supplementary figures and images for: Analysis of LINE1 Retrotransposons in Huntington’s Disease
Source: Front Cell Neurosci. 2022 Jan 14;15:743797. doi: 10.3389/fncel.2021.743797 (PMC8795916; doi:10.3389/fncel.2021.743797)

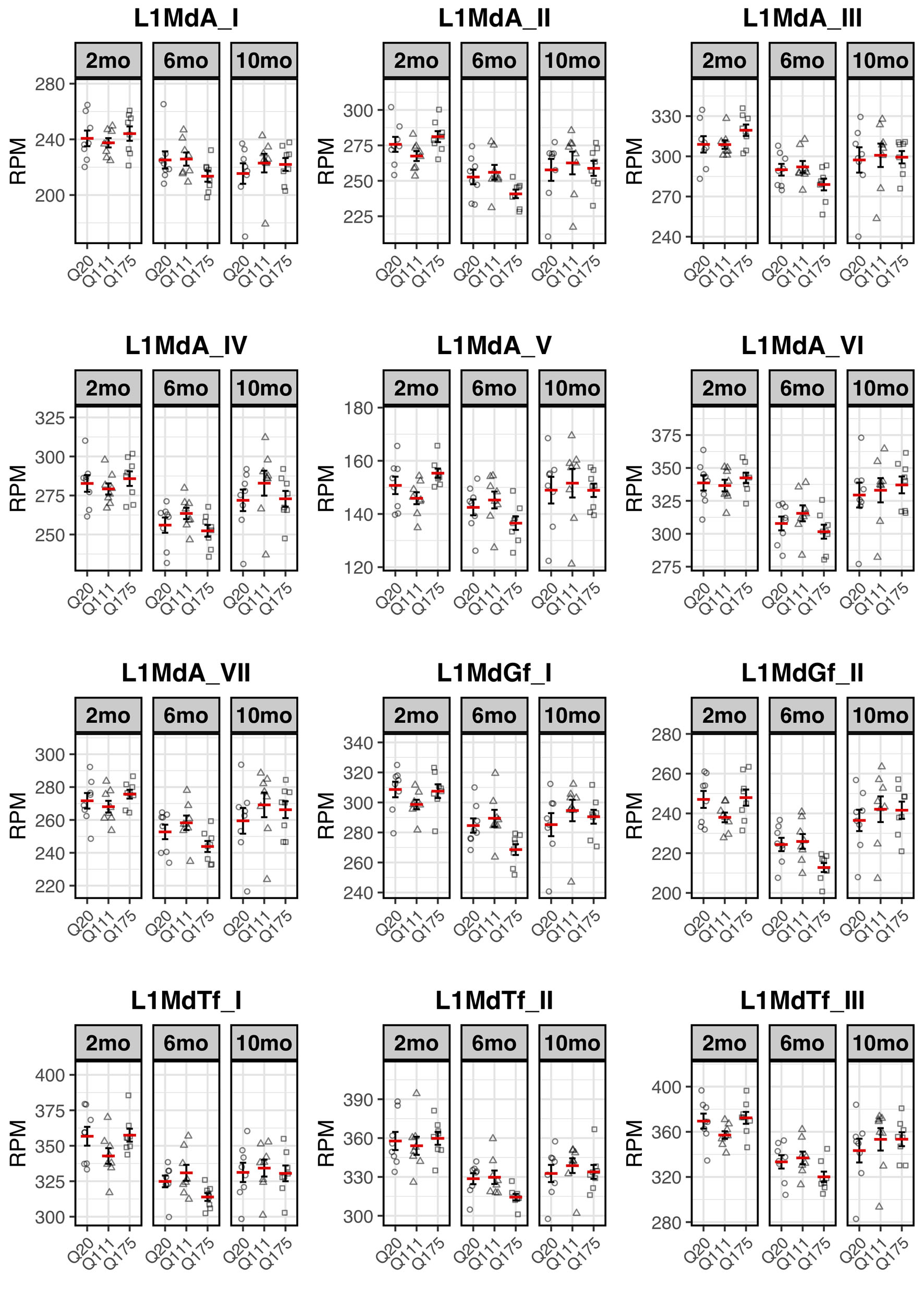

Supplement: Supplementary Figure 1 — Expression of the 7 L1-A (L1MdA), 2 L1-Gf (L1MdGf), and 3 L1-Tf (L1MdTf) in the mouse cortex. L1 expression is reported as number of mapped reads normalized on the total number of reads of each sample (RPM) for Q20 (control) (circle), Q111 (triangle) and Q175 (square) in 2-month-old mice (2 mo—left panel), 6-month-old mice (6 mo—middle panel), and 10-month-old mice (10 mo—right panel). Horizontal red segment represents mean, error bars report mean ± sem (standard error of the mean). *FDR < 0.05, **FDR < 0.01, ***FDR < 0.001. [file Image_1.JPEG]

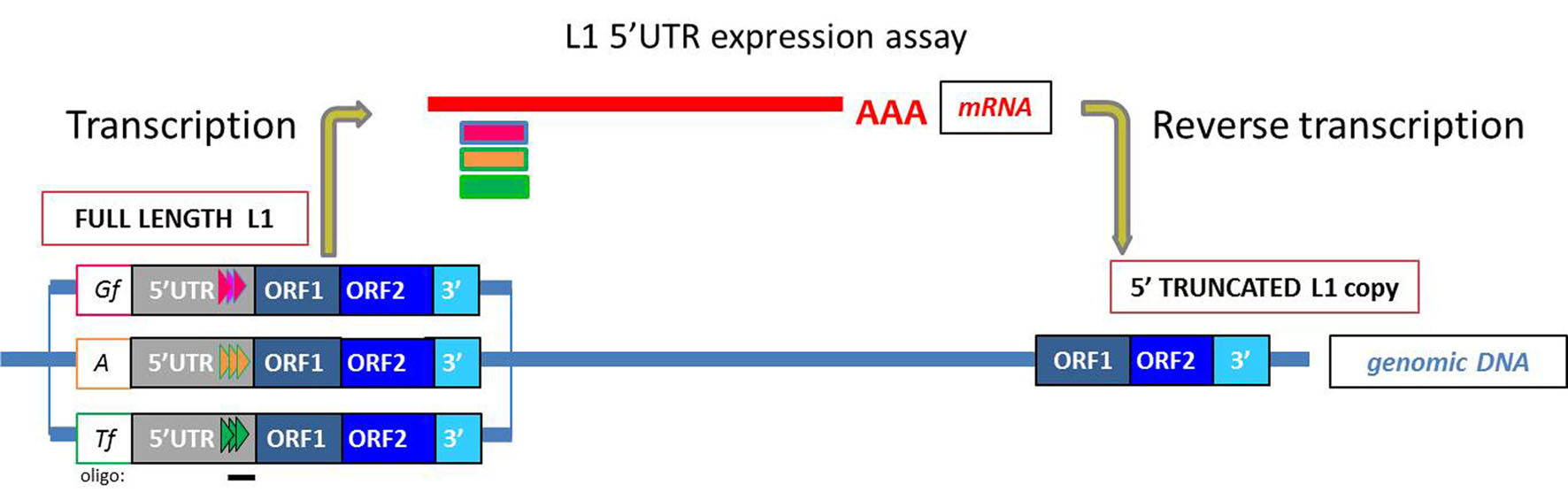

Supplement: Supplementary Figure 2 — Schematic representation of 5′UTR L1 Taqman assay on full-length L1s. Purple, yellow and green boxes indicate, respectively, Gf, A and Tf Taqman probes, able to detect only full-length L1 mRNAs. The 5′UTR monomers of full-length mouse L1 elements are represented by triangles and the black bar below indicates the position of primers and Taqman probes specifically designed for each monomeric L1 region. [file Image_2.JPEG]
